# Vanadium improves memory and spatial learning and protects the pyramidal cells of the hippocampus in juvenile hydrocephalic mice

**DOI:** 10.3389/fneur.2023.1116727

**Published:** 2023-02-09

**Authors:** Omowumi Moromoke Femi-Akinlosotu, Funmilayo Eniola Olopade, Jane Obiako, James Olukayode Olopade, Matthew Temitayo Shokunbi

**Affiliations:** ^1^Developmental Neurobiology Laboratory, Department of Anatomy, College of Medicine, University of Ibadan, Ibadan, Nigeria; ^2^Neuroscience Unit, Department of Veterinary Anatomy, Faculty of Veterinary Medicine, University of Ibadan, Ibadan, Nigeria; ^3^Division of Neurological Surgery, Department of Surgery, University of Ibadan, Ibadan, Nigeria

**Keywords:** hydrocephalus, vanadium, hippocampus, pyramidal neurons, pyknotic index

## Abstract

**Background:**

Hydrocephalus is a neurological condition known to cause learning and memory disabilities due to its damaging effect on the hippocampal neurons, especially pyramidal neurons. Vanadium at low doses has been observed to improve learning and memory abilities in neurological disorders but it is uncertain whether such protection will be provided in hydrocephalus. We investigated the morphology of hippocampal pyramidal neurons and neurobehavior in vanadium-treated and control juvenile hydrocephalic mice.

**Methods:**

Hydrocephalus was induced by intra-cisternal injection of sterile-kaolin into juvenile mice which were then allocated into 4 groups of 10 pups each, with one group serving as an untreated hydrocephalic control while others were treated with 0.15, 0.3 and 3 mg/kg i.p of vanadium compound respectively, starting 7 days post-induction for 28 days. Non-hydrocephalic sham controls (*n* = 10) were sham operated without any treatment. Mice were weighed before dosing and sacrifice. Y-maze, Morris Water Maze and Novel Object Recognition tests were carried out before the sacrifice, the brains harvested, and processed for Cresyl Violet and immunohistochemistry for neurons (NeuN) and astrocytes (GFAP). The pyramidal neurons of the CA1 and CA3 regions of the hippocampus were assessed qualitatively and quantitatively. Data were analyzed using GraphPad prism 8.

**Results:**

Escape latencies of vanadium-treated groups were significantly shorter (45.30 ± 26.30 s, 46.50 ± 26.35 s, 42.99 ± 18.44 s) than untreated group (62.06 ± 24.02 s) suggesting improvements in learning abilities. Time spent in the correct quadrant was significantly shorter in the untreated group (21.19 ± 4.15 s) compared to control (34.15 ± 9.44 s) and 3 mg/kg vanadium-treated group (34.35 ± 9.74 s). Recognition index and mean % alternation were lowest in untreated group (*p* = 0.0431, *p*=0.0158) suggesting memory impairments, with insignificant improvements in vanadium-treated groups. NeuN immuno-stained CA1 revealed loss of apical dendrites of the pyramidal cells in untreated hydrocephalus group relative to control and a gradual reversal attempt in the vanadium-treated groups. Astrocytic activation (GFAP stain) in the untreated hydrocephalus group were attenuated in the vanadium-treated groups under the GFAP stain. Pyknotic index in CA1 pyramidal layer of untreated (18.82 ± 2.59) and 0.15mg/kg vanadium-treated groups (18.14 ± 5.92) were significantly higher than control (11.11 ± 0.93; *p* = 0.0205, *p* = 0.0373) while there was no significant difference in CA3 pyknotic index across all groups.

**Conclusion:**

Our results suggest that vanadium has a dose-dependent protective effect on the pyramidal cells of the hippocampus and on memory and spatial learning functions in juvenile hydrocephalic mice.

## Introduction

Vanadium, a naturally occurring ubiquitous element with atomic number 23 (1, 2), well known for its toxicity, can be found in trace amounts in certain foods, beverages and water (1, 3). Some reports suggest that vanadium is essential for certain plants, bacteria, algae, fungi and nitrogen fixing microorganisms, chicks (4) and mammals such as rats and goats (1, 4–6). Although no sufficient evidence concludes its essentiality for humans, it is being used and sold as dietary supplements for athletes and bodybuilders (1, 6, 7). Intake of ≤ 4500 μg/day is considered safe and adequate for human consumption (1). Despite its well documented toxic effects, studies recently have explored its restorative potentials expressed at lower concentrations and discovered that although vanadium crosses the blood-brain barrier (BBB) causing lesions to different parts of the brain (8), it can serve as a therapeutic agent in BBB disruptions (3), improve learning, memory and cognitive functions (3, 9) and provide neuroprotection in spinal cord injury, traumatic brain injury, and stroke (10–15).

It has been suggested that the vanadium complex (oxovanadium hydropolypyrazolylborate complex) with relatively lower toxicity can improve the learning and memory ability in diabetic mice Chen et al. (9). Suarez (3) exposed rats to vanadium *via* food mash containing 0.05 mg/kg vanadium and showed cognitive improvement compared to control rats who received no vanadium in their diet, suggesting that vanadium has a beneficial effect in cognition. Walker and Xu (13) demonstrated that treatment with vanadium compounds promoted white matter and myelin sparing, stability and remyelination as well as protected and increased oligodendrocytes and their precursor cells. Vanadium has also been reported to possess some antioxidant and anti-inflammatory properties (12, 16–18).

Hydrocephalus, a relatively common neurological condition in children, occurring in 145 per 100,000 live births in Africa (19). It involves accumulation of cerebrospinal fluid (CSF) in the ventricles of the brain causing structural injury to the surrounding the white and gray matter including the hippocampus. Its clinical presentation varies with chronicity and often shows cognitive dysfunction (20). The hippocampus, an important component of the limbic system, mainly responsible for learning and memory (21) also plays a key role in spatial navigation. Damage to the hippocampus due to hydrocephalus may cause loss in its cognitive, learning and memory functions (20, 22). Hydrocephalus has been known to cause inflammation, myelin disruption, axonal damage within the periventricular white matter and loss in oligodendrocytes (20, 23–25). The current standard definitive treatment for established hydrocephalus is surgical diversion of CSF, but this is expensive and associated complications such as shunt obstruction and infections. Recently, research has been geared toward exploring alternative therapeutic interventions that may be able to mitigate the structural injury and corresponding neurological deficits associated with hydrocephalus (26, 27).

Limited information is available on the effect of vanadium on the brain in hydrocephalic state. In this study, we investigated: (a) structural alterations of the pyramidal neurons in the CA1 and CA3 of the hippocampus and the changes in neurobehavior of juvenile hydrocephalic mice; (b) examined the impact of different doses of vanadium compound on these changes.

## Materials and methods

### Animals

Three-week old mice were obtained from the Central Animal House of the Faculty of Basic Medical Sciences, University of Ibadan. All experiments were approved by the Ethical Review Board of the University of Ibadan with assigned number UI-ACUREC/026-0421/20 and were carried out both in the Central Animal House and Postgraduate students' laboratory of the Department of Anatomy.

Hydrocephalus was induced by injecting sterile kaolin suspension (0.02 ml of 250 mg/mL) into the cisterna magna of the experimental mice, under anesthesia 0.2 ml of ketamine injected intraperitoneally while control mice received sham injections, also under anesthesia. The hydrocephalic animals were batched into 4 groups of 10 mice each: an untreated hydrocephalus group which served as the negative control, a low dose (0.15 mg/kg) vanadium group, an intermediate (0.3 mg/kg) dose vanadium group and a high dose (3 mg/kg) vanadium group. The vanadium treated animas received powdered sodium metavanadate (Santa Cruz Biotechnology Inc. Chem Crux Dallas, Texas) for 28 days beginning 1 week (7 days) post induction of hydrocephalus. The animals were weighed and treated at a 72-h intervals.

### Behavioral tests

Behavioral tests for learning and memory were performed 30–35 days post-induction using Modified Morris Water Maze, Y maze and Object Recognition tests.

### Modified Morris water maze test

The Morris water maze tests hippocampal-dependent spatial learning and memory in rodents (28). It consists of a circular pool of opaque water in a container (120 cm in diameter, 30 cm in height) with a hidden circular escape platform (12 cm in diameter) 1 cm below the water level where the mouse must learn its location using contextual and visual cues. The pool was marked North, South, East and West and the platform placed in a particular spot throughout the duration of the trials. Each mouse was dropped into the tank, head facing the wall of the tank and allowed to freely swim, during which time were expected to search for the platform. The time (in seconds) it takes the animal to find the platform was recorded. If it failed to find the platform in 120 s, the mouse was guided to the platform and allowed to stay there for 15 s. Each mouse went through four trials per day for three consecutive days. This tested the learning ability of the individual mouse (29). On the fourth day, a single probe trial was given to test the mice' spatial memory in the water maze while the platform was removed. The mice' memory of the initial location of the platform was measured by the time spent in the target quadrant and the number of times it crossed the island zone where the platform was initially located.

### Novel object recognition test

The novel object recognition test (NORT) is a relatively fast and efficient behavioral test used to investigate various aspects of learning and memory in mice (30). This test assesses cognition, specifically spatial memory and discrimination, in rodent models of CNS disorders. It has been used as a model of hippocampal-dependent memory. The test is based on the spontaneous tendency of rodents to spend more time exploring a novel object than a familiar object; their innate preference for novelty (31) and it measures the percentage novel object (% NOR) which is called the recognition index; an index of memory retention (32, 33). The tests span through a period of 3 days: habituation day, training day, and testing day. On the first day (habituation day), the mouse was placed in the middle of the open arena, a white painted wooden box measuring 72 cm by 72cm and allowed to freely explore for 5 min. The second day (24 h later; training day), 2 identical objects were placed in an equidistant, diagonal location and the mouse was placed in the middle of the open arena and allowed to explore the 2 identical objects for 5 min. Lastly, on the test day, one of the training objects was replaced with a novel object. The time spent exploring both familiar and novel objects were recorded. Exploration recorded were times the mouse's nose is pointed toward the object and within 2–3 cm of the object, with active sniffing. Time spent sitting on objects without any indication of active exploration was not recorded. The objects and the arena were cleaned after every individual mouse session with 70% alcohol to minimize odor cues.

### Y-maze

The Y-maze was used to assess short term memory in mice. Spontaneous alternation, a measure of spatial working memory, was assessed by allowing mice to explore all three arms of the maze. This test is driven by an innate curiosity of rodents to explore previously unvisited areas (34). Each mouse was placed in the center of the maze and allowed to explore the arms for 5 min. All arm entries were recorded accordingly except the arm that the mouse entered when it was first placed in the maze.

Spontaneous alternation will be calculated thus:


(1) $$\begin{array}{l} \%\text{ alternation}=\frac{spontaneous\;alternation}{total\;number\;of\;enteries-2}\;x\;100 \end{array}$$

(34, 35).

The maze was cleaned with 70% alcohol to eliminate possible bias due to odor cues.

## Animal sacrifice and histology

The mice were anesthetized using 0.2 ml of ketamine and then transcardially perfused with 10% neutral buffered formalin after the last neurobehavioral test was carried out on day 35 post-hydrocephalus induction. The mice brains were then removed and fixed in the same fixative, dehydrated in graded series of alcohol, cleared in xylene, infiltrated in molten paraffin wax and embedded in paraffin wax. Coronal sections at the level of optic chiasma were obtained from the tissues blocks and sectioned at 7 microns thickness for cresyl violet and immunohistochemical staining.

## Histomorphometric analysis

Photomicrographs of the brain sections were taken with the aid of a digital light microscope (Leica, Germany) at × 40 objective lens. The cresyl violet stained coronal histological sections were used for assessing neuronal and pyknotic cells using computerized image analyzers (Image J v1.53e). For each group, average neuronal counts were obtained by counting four serial coronal sections using a standardized square of 2400 mm^2^. The pyknotic index was (36) calculated thus:


(2) $$\begin{array}{l} \text{Pyknotic Index}\left(\text{PI}\right)=\frac{Pyknotic\;neurons}{total\;number\;of\;neurons}\;x\;100 \end{array}$$

(36).

## Immunohistochemistry

According to Folarin et al. (8), the paraffin sections were dewaxed, rehydrated and immersed in distilled water, then put in a 10 mM citrate buffer (pH = 6.0) for 25 mins for the Antigen retrieval process, with subsequent endogenous peroxidase blocking in 3% H_2_O_2_/methanol for 20 mins. The sections were blocked in 2% PBS milk for 1 h and probed with the following antibodies: anti-NeuN Rabbit monoclonal antibody for neuronal morphology (1:3000, Abcam, Cambridge, MA, USA) and anti-GFAP Rabbit Polyclonal antibody for astrocytic morphology (1:1000, Dako, Denmark) at 40°C overnight. After washing, the sections were incubated for 1 h at room temperature in biotinylated secondary antibodies (diluted 1:200; Vector Labs, USA), after which the sections were incubated in avidin biotin-peroxidase solution according to manufacturer's protocol (ABC kit, Vectastain, Vector Labs, USA) for 1 h which then reacted with 3,30-diaminobenzidine as chromogen.

## Data analysis

Quantitative data from the scores in the behavioral tests, neuronal count and pyknotic count were presented as mean ± SEM. All data sets were analyzed by GraphPad Prism 8.4 using Analysis of Variance test to compare the means of all groups and Tukey's multiple comparison *post-hoc* test to compare within the groups. The statistical significance was set at *p* < 0.05.

## Results

### Physical observation

**Figure 1:**
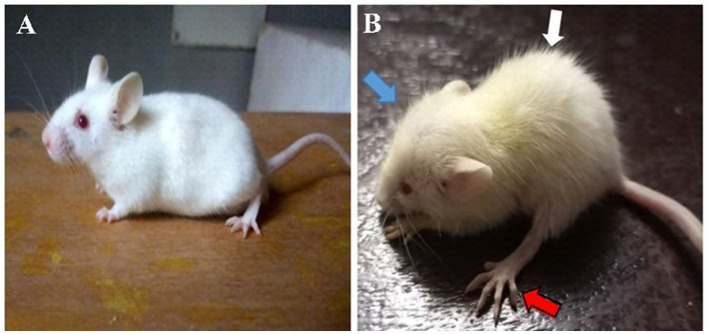
Photographs of control **(A)** and hydrocephalic mice **(B)**. Hydrocephalic mouse presents with a dome-shaped head (blue arrow), hunched back (white arrow), flat placement of limb (red arrow) and scruffy fur compared to the control mouse.

**Figure 2:**
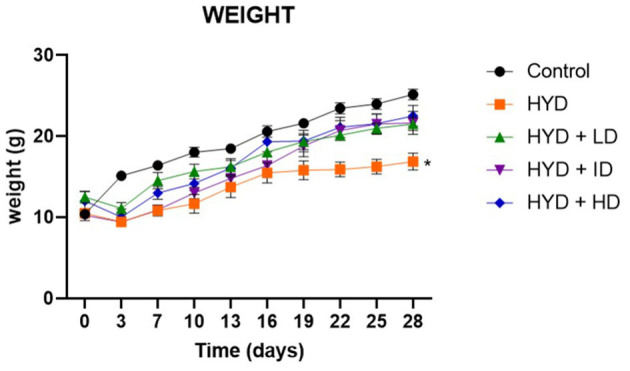
Line graph showing the body weight progression of all groups over a period of 28days. (Control; HYD, untreated hydrocephalus; HYD+LD, hydrocephalus + low dose vanadium; HYD+ID, hydrocephalus + intermediate dose vanadium; HYD+HD, hydrocephalus + high dose vanadium; ^*^p < 0.05; *p* = 0.0264).

The hydrocephalic mice exhibited a reduction in activity, food and water intake; developed an enlarged dome-shaped head, a hunched back, unsteady gait due to flat limb placement and scruffy fur due to impaired grooming abilities (Figure 1). There was an initial reduction in body weights of the hydrocephalic mice in the first 3days post- induction, followed by a slow but steady weight gain recorded over the remaining period of the study. When compared to the control, the body weights of the untreated hydrocephalus group were significantly lower (*p* < 0.05); the vanadium treated groups showed better body weight gain recovery when compared with the untreated hydrocephalus group (Figure 2).

## Behavioral tests

### Morris water maze

**Figure 3:**
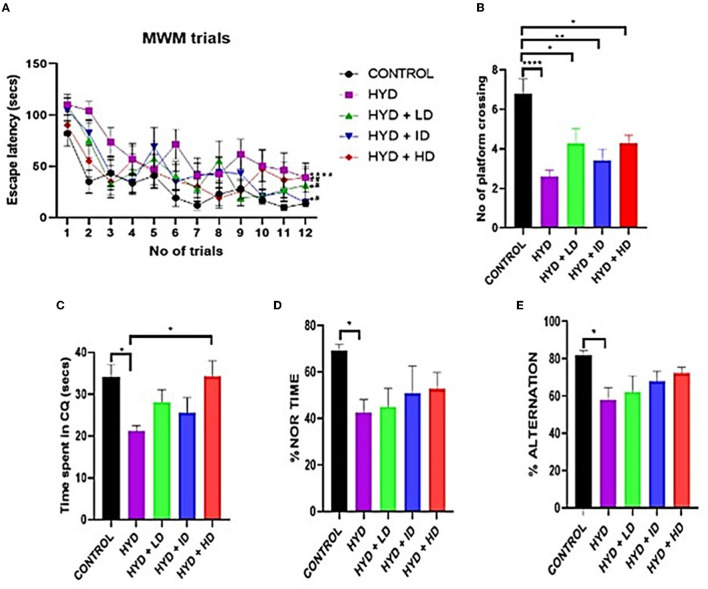
Behavioral tests. **(A)** Line graph showing the escape latency period of control and experimental animals. **(B–E)** Bar charts showing the number of platform crossing; time spent in the correct quadrant; % novel object recognition time and % alternation of control and experimental mice. (Control n = 10; HYD n = 10; HYD+LD n = 7; HYD+ID n = 7; HYD+HD n = 7. *compared to control; ^#^compared to hydrocephalic group).

During the training sessions, all mice had a gradually decreasing latency to locate the hidden platform with each trial except for the untreated hydrocephalus group, although all the experimental mice learned much slower than their age-matched control mice suggesting an impairment in learning abilities in the experimental group. The time it took the untreated hydrocephalic group (*t* = 62.06 s; *p* < 0.0001), low dose (*t* = 45.30 s; *p* < 0.05) and intermediate dose (*t* = 46.50 s; *p* < 0.05) vanadium treated groups to find the hidden platform were significantly longer than the control (*t* = 29.91 s) however there was an improvement in learning abilities of the vanadium treated groups with the significantly shorter escape latency periods of the low dose (*t* = 45.30 s; *p* < 0.05), intermediate dose (*t* = 46.50 s; *p* < 0.05) and high dose vanadium treated groups (*t* = 42.99 s; *p* < 0.01) when compared to the untreated hydrocephalus group (Figure 3A). For the probe trial on the 4th day, the control group crossed the area where the platform was originally placed in a significantly higher number of times than the untreated hydrocephalus group (*n* = 2.600; *p* < 0.0001), low dose (*n* = 4.286; *p* < 0.05), intermediate dose (*n* = 3.429; *p* < 0.01) and high dose (*n* = 4.286; *p* < 0.05) vanadium treated groups (Figure 3B). Similarly, the untreated hydrocephalus group spent the shortest time in the correct quadrant where the hidden platform was originally located (*t* = 21.19 s) when compared to the other groups, which was significantly lower than the time spent by the control (*t* = 34.15 s; *p* < 0.05) and the high dose vanadium treated group (*t* = 34.35 s; *p* < 0.05) (Figure 3C). This suggests an impairment in memory functions in the hydrocephalus group and a recovery in the treated groups.

### Novel object recognition

The untreated hydrocephalus group showed the least memory retention ability compared to the other groups. The untreated hydrocephalus group showed significantly lower % NOR time when compared to control (*p* = 0.0324) (Figure 3D).

### Y-maze

The untreated hydrocephalus group had the lowest percentage alternation compared to the other groups and it was especially significantly lower than the control group (*p* = 0.0158) (Figure 3E).

## Histology

**Figure 4:**
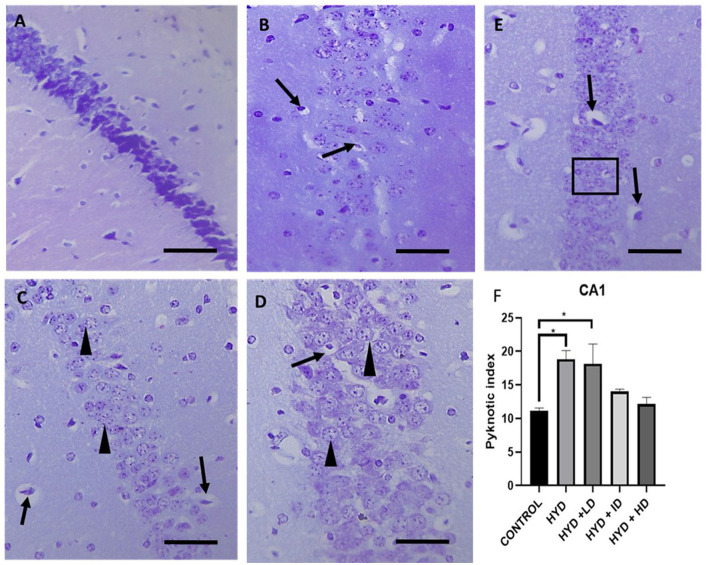
Photomicrograph of Cresyl stained CA1 region of the hippocampus of control and experimental mice. **(A)** Control, **(B)** hydrocephalus only (HYD), **(C)** hydrocephalus + low dose vanadium (HYD+LD), **(D)** hydrocephalus + intermediate dose vanadium (HYD+ID), **(E)** hydrocephalus + high dose vanadium (HYD+HD). Note the cells of the control compared to those of the other groups, also the cells of the vanadium treated groups show cell recovery (as indicated by the normal cells) and less pyknotic cells than the hydrocephalus only group, and **(F)** bar chart showing the pyknotic index of the CA1 region of the hippocampus of the control and experimental groups. The pyknotic index of the hydrocephalic group and low dose vanadium treated group were significantly higher compared to that of the control [arrows, pyknotic neurons; arrowheads and inserted box in **(E)**, normal neurons; Scale bar, 4μm].

**Figure 5:**
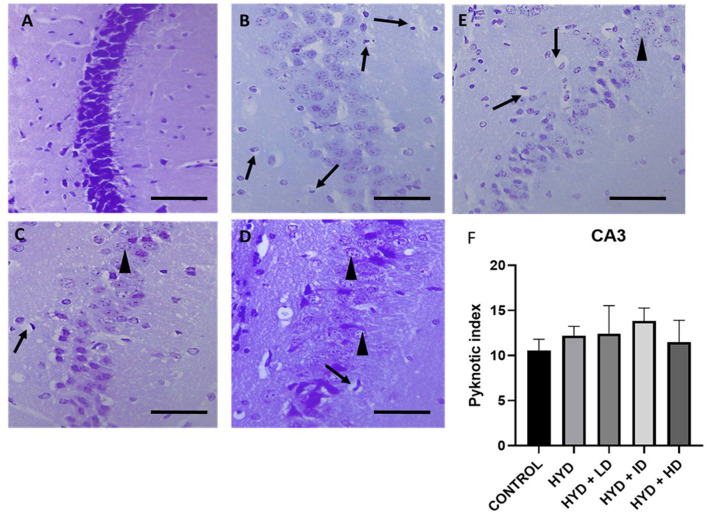
Photomicrograph of Cresyl stained CA3 region of the hippocampus of control and experimental mice. **(A)** Control, **(B)** hydrocephalus only (HYD), **(C)** hydrocephalus + low dose vanadium (HYD+LD), **(D)** hydrocephalus + intermediate dose vanadium (HYD+ID), **(E)** hydrocephalus + high dose vanadium (HYD+HD), and **(F)** bar chart showing the pyknotic index of the CA3 region of the hippocampus of the control and experimental groups. There was no significant difference in the pyknotic index in the CA3 region among all the groups (arrows, pyknotic neurons; arrowheads, normal neurons; Scale bar, 4μm).

The cresyl stained sections revealed a tight compact layering of the hippocampal pyramidal cells with a robust neuronal population and well-defined nuclei in the CA1 and CA3 areas in the control group (Figures 4, 5). The sections in the untreated hydrocephalic group revealed a mild scattering of the pyramidal cell layer with loss of cells compared to the control. The pyramidal layers of the vanadium-treated sections were also in a state of disarray and not as compact and characteristically aligned as in the sections, but appeared to be recovering especially in the CA1 region when compared with the untreated hydrocephalus group (Figure 4). The high dose vanadium treated sections showed the best recovery attempt with the arrangement of the cells being near as compact as observed in the control sections (Figure 4E). The distinctively pyramidal shape of the neuronal cell body in the control sections were evident in the control sections while there were dark, shrunken pyknotic neurons with vacuolations and abnormal chromatin clumps alongside altered shape of pyramidal cell bodies in the hydrocephalic sections, although the cells of the vanadium-treated groups appeared to be regenerating when compared to the untreated hydrocephalus group (Figures 4, 5). The pyknotic pyramidal cells were particularly evident in the CA1 region of the hippocampus (Figure 4). The CA3 region also revealed a number of pyknotic neurons but it was not significantly different from the control (Figure 5).

## Neuronal count

The pyknotic index (PI) was significantly increased in the CA1 region of the hippocampus in the untreated hydrocephalus group (*p <* 0.05) and low dose vanadium-treated group (*p <* 0.05) when compared to the control group but not in the intermediate and high dose vanadium-treated groups (Figure 4F). There was no statistically significant difference in the PI of CA3 region of the experimental groups when compared to control (Figure 5F).

## Immunohistochemistry

**Figure 6:**
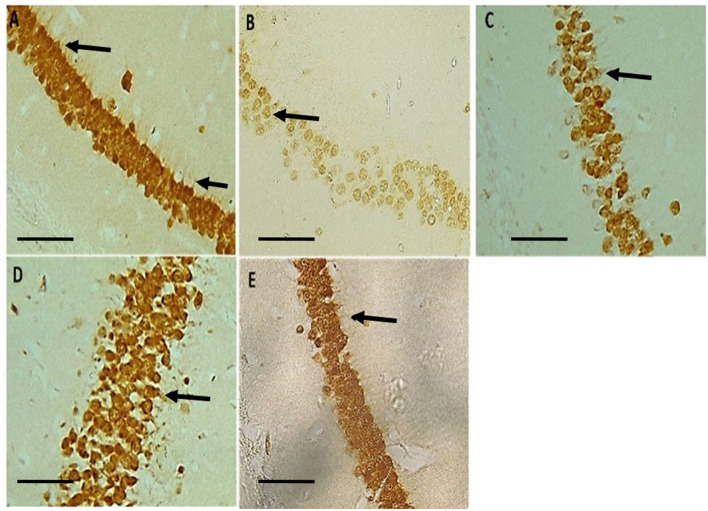
Photomicrograph of Neu-N immuno-stained CA1 region of the hippocampus of control and experimental mice revealing a loss in apical dendrites of the pyramidal cells (black arrows) in untreated hydrocephalus group relative to control and a gradual reversal attempt affected in the vanadium-treated groups. **(A)** Control, **(B)** untreated hydrocephalus, **(C)** hydrocephalus + low dose vanadium, **(D)** hydrocephalus + intermediate dose vanadium, and **(E)** hydrocephalus + high dose vanadium; (Scale bar, 4μm).

**Figure 7:**
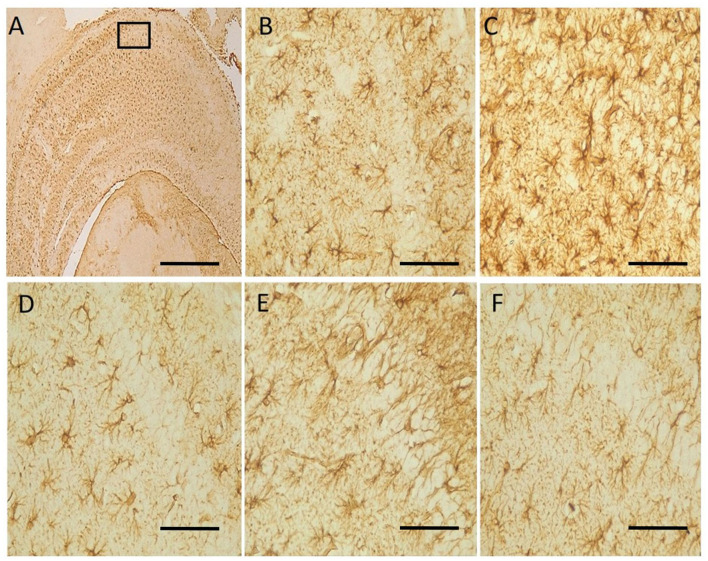
Photomicrograph of anti-GFAP immuno-stained CA1 region of the hippocampus of control and experimental mice. Untreated hydrocephalus group **(C)** revealed astrocytic activation identified by thickened cell body with extended processes and dense population relative to control while vanadium treatment showed an attempt at mitigating the effect of hydrocephalus relative to the untreated group **(B–F)**. **(A)** Control [insert box: area magnified in **(B–F)**], **(B)** control, **(C)** untreated hydrocephalus, **(D)** hydrocephalus + low dose vanadium, **(E)** hydrocephalus + intermediate dose vanadium, and **(F)** hydrocephalus + high dose vanadium; [Scale bar, **(A)** 50μm; **(B–F)** 4μm].

Neu-N immunolabelling in the hippocampus revealed altered morphology of pyramidal cells with a loss in apical processes and neuronal population of the pyramidal cells in untreated hydrocephalus group relative to control and a gradual reversal attempt observed in the vanadium-treated groups (Figure 6). There was increase astrocytic activation in the untreated hydrocephalus group suggesting an increase in neuroinflammation. This expression was seen to be reduced in the vanadium-treated groups (Figure 7).

## Discussion

In this study, the induction of hydrocephalus in juvenile mice led to reduced body weight, poor performance in the neurobehavioral tasks, disorganization of hippocampal pyramidal layers, increased pyknotic index of the CA1 pyramidal cells of the hippocampus, and loss in apical dendrites and neuronal population. Vanadium treatment, however, mitigated the effects of hydrocephalus and showed a dose-dependent recovery from these anomalies. There is no ideal animal model for hydrocephalus, due to the multifactorial nature of its pathophysiology and the fact that no model can fully mimic the human condition yet (37–40). The most recognized method of effectively inducing hydrocephalus in animals especially rodents is by intracisternal injection of sterile kaolin (aluminum silicate) which was used in this study, which has been known to cause an inflammatory reaction and meningeal scarring leading to an obstructive hydrocephalic condition (23, 38, 41). This model has contributed significantly to the understanding of the pathogenesis of hydrocephalus (41). The brain of juvenile mice (three-week old) is developmentally similar to that of a human infant (23, 42), Thus, cisternal kaolin injection in juvenile rodents is a reasonable model for studying infantile hydrocephalus (20).

In the quest for finding non-surgical means of treating hydrocephalus, several experimental methods have been explored including use of intraperitoneal injection of deferoxamine, a chelating agent that binds free ferric iron (Fe^3+^) which was found to have neuroprotective and anti-fibrotic properties in experimental animals with hydrocephalus (43–45). Anti-inflammatory, anti-oxidative, neuronal and axonal protection interventions by use of intraperionteal injection of Minocycline (46–48), epigalloctechin gallate from green tea and N-acetylcystein (27, 49), melatonin (50, 51), memantine (52) and caffeine (23) have been reported to offer neuroprotection in experimental hydrocephalic conditions. Vanadium compound which has been reported to possess antioxidant properties and which promotes neuroprotection and cell recovery in neurotrauma and neurodegenerative disorders (12, 17, 53, 54) has not been previously studied for possible beneficial effect in hydrocephalus.

In our study, the kaolin-induced animals presented with dome-shaped head, hunched back, flat placement of limbs as well as ill-groomed scruffy fur, confirmatory of successful induction of hydrocephalus, as described in previous studies (5, 20, 26, 41, 55). There was loss of body weights of the hydrocephalic mice within the first few days post induction, one of the earliest signs of development of hydrocephalus, possibly due to growth delay, loss in appetite and overall reduction in feeding (41, 55–57). Subsequently, there was a gradual recovery of body weight in all experimental groups but the weight gain gradient of the hydrocephalus only group was still significantly lower than that of the control group. The vanadium-treated groups however, had a better weight gain gradient when compared to the untreated hydrocephalus group, possibly because vanadium has been reported to have growth factor mimetic or enhancing effects (58, 59) and it's used as dietary supplements for athletes and bodybuilders (1, 6, 7), as well as in the treatment of malnutrition (2, 60).

Hydrocephalus is often characterized by loss learning and memory disabilities It has been suggested that this is due to the vulnerability of the hippocampus to injury in hydrocephalus in humans and in animal models (36, 41, 52). The untreated hydrocephalus group had the most learning disability as seen by the significantly longer escape latency periods during the trials, similar to observations by Olopade and Shokunbi (28), Chen et al. (20), and Olopade et al. (55), and suggestive of damage to the hippocampus and/or its neural circuitry (20). The low dose and intermediate dose vanadium-treated groups had a significantly poorer performance in finding the hidden platform compared to the control groups, however, all vanadium-treated groups had significantly better learning performances compared to the untreated hydrocephalus group corroborating the report by Dyer and de Butte (61) in which vanadium treated rats exhibited significantly shorter latency times to find the hidden platform. This suggests that vanadium improves the learning deficit of hydrocephalic mice which was similar to earlier findings by He et al. (62) who suggested that vanadium treatment ameliorates learning disability in Alzheimer's Disease (AD) mice model. In memory consolidation analyzed on the MWM test day, all vanadium-treated groups showed an improvement in recollection memory when compared to the untreated hydrocephalus group. Although there was no significant difference in the number of platform crossing of the vanadium-treated groups as observed by Di Curzio et al. (26) that reported oral antioxidant therapy with Canola oil did not provide significant impact on the Morris Water Maze tests in juvenile hydrocephalic rats induced with Kaolin post-treatment as well as 60: vanadium had no significant impact during the probe trial. The high dose vanadium-treated group spent a significantly longer time in the correct quadrant than the untreated group; although Folarin et al. (63) had earlier reported that high doses of vanadium produced spatial memory deficits in animals with no pathology (63). Spatial working memory recorded as spontaneous alternation and recognition memory using the index of memory retention was tested using the Y-maze and Novel Object Recognition tests respectively. We observed a significant loss in spatial working memory and recognition index in the untreated hydrocephalus group compared to the control group corresponding with previous reports (64, 65). The vanadium treated groups however, had a tendency toward improvement in percentage alternation and percentage novel object recognition time when compared to control but did not reach significant levels. Dyer (66) opined that chronic low dose vanadium administration did not have an effect on the total object recognition time. There is however scanty evidence in literature that has reported improvement in working memory with vanadium use suggesting that the effect of vanadium on cognition and the mechanism by which it acts has not been fully explored.

Numerous pyknotic cells with dark shrunken nuclei, vacuolations and abnormal chromatin clumps were observed in the untreated hydrocephalus group with a more significant increase in the CA1 region than the CA3 region of the hippocampus when compared to the control group. This is similar to the study by Shokunbi et al. (41) which reported a significant increase in pyknotic cells in the CA1 region of the 7 day post-hydrocephalus induced mice with no significant impact on the CA3 region due to the vulnerability of the CA1 region to hypoxia and ischemia caused by hydrocephalus causing a more pronounced pyramidal cell damage in this area. Chen et al. (20) reported that increased pyknotic neurons in the CA1 region was due to the loss of its excitatory connectivity. Thus, impairment in learning and memory in the hydrocephalic mice could be attributed to the significantly increased number of pyknotic neurons (67). The pyknotic index in the CA1 region of the low dose vanadium-treated groups was also significantly higher than the control group; this could explain their poor performance in the behavioral tests in comparison to those of the intermediate and high doses. In contrast to these results, previous studies have reported that vanadium exhibits neuroprotective qualities at low doses (12–15, 68–70) and learning and memory deficits at high doses (71–73). Chen et al. (9) suggested that vanadium (oxovanadium hydropolypyrazolylborate complex) at relatively lower doses can improve the learning and memory ability of diabetic mice. Suarez (3) reported that rats exposed to vanadium *via* food mash containing 0.05 mg/kg vanadium showed cognitive improvement compared to control rats who received no vanadium in their diet, suggesting that vanadium has a beneficial effect in cognition in rats exposed to vanadium through their food. Han et al. (74) however, reported an amelioration in cholinergic cell loss in the septum and a reversal in the hippocampal long term potentiation and spatial memory impairments in olfactory bulbectomized mice with an administration of 0.5–1 mg/kg/day of vanadium compound, a dose higher than the intermediate dose in our study. Although studies like Folarin et al. (63) have reported a deficit in spatial memory 3 months after exposure to 3 mg/kg of vanadium and Adebiyi et al. (75) reported that a daily dose of 3 mg/kg of sodium metavanadate induced learning and memory deficit as a result of neurodegeneration of the hippocampus. 75 reported an impairment in spatial memory only at the highest dose in rats treated with 15, 20, or 25 mg/kg/day for 2 weeks.

Brain functions are ultimately defined by neuronal integrity, connectivity and functions (76), and the pyramidal neurons being major output neurons of the hippocampus have neural connections and circuitry which control cognition, learning and memory functions (20). Therefore, destruction of these neural circuitry may lead to cognitive dysfunctions and hydrocephalus has been implicated in these destructions (20, 41). Apart from the neural circuitry, hydrocephalus has been reported to cause alterations in the morphology of the hippocampal pyramidal cells (36, 41) and neuroinflammation through increased activation of astrocytes (77–79). GFAP-immunoreactivity has been studied using experimental hydrocephalic animals (55, 80–82). Reactive astrogliosis is common in hydrocephalus (80). Astrogliotic scarring and reduced exchange between CSF and interstitial fluid, as well as CSF reabsorption through the meningeal lymphatic vessels have been implicated in germinal matrix hemorrhage (GMH) as a result of rupture of the immature thin-walled blood vessels (83). Stretching and compression of the brain tissue caused by the enlarged ventricles can abet the proliferation of astrocytes, damaging the connectivity pathways, interrupting cellular metabolism, disrupt cerebral blood flow and ultimately leading to cellular death. This might account for the neurologic and motor deficits experienced in hydrocephalic states. The Neu-N immunostaining revealed a loss in apical processes and neuronal population as well as a disruption of the pyramidal cell layer of the untreated hydrocephalus groups compared to the control, thus causing a loss in their characteristic alignment as reported by previous studies (20, 36, 41, 67). Intriguingly, our study reports a dose-dependent attempt at recovery in the neurobehavioral parameters assessed and in the arrangement of the pyramidal cell layers, number of pyknotic cells and astrocytic reaction (GFAP immunostaining) in the vanadium-treated group especially in the CA1 region, with the high dose (3 mg/kg) showing the best recovery relative to the untreated group. This is similar to a finding by Sury et al. (84) who reported significant attenuation of hippocampal apoptosis with repetitive 4-h intraperitoneal doses of 2 mg/kg bpV. Vanadium has been reported to downregulate inflammatory reactions (16) and reduce brain damage through the deletion or down-regulation of phosphatase and tensin homolog (PTEN) and an up-regulation of phosphoinositide-3 kinase (PI3K)/protein kinase B (Akt)/mammalian target of rapamycin (mTOR) pro-survival pathway, which is known to enhance cell survival, growth and regenerative ability of adult corticospinal neurons and important for controlling voluntary movement necessary for mouse exploratory behavior action (12–15, 59, 68). This could also explain the recovery in the learning and memory functions observed by the general performance of the vanadium-treatment groups when compared to the untreated group. Interestingly, Hasegawa et al. (85) reported that animals with subarachnoid hemorrhage (SAH) treated with 3 mg/kg and 10 mg/kg of sodium orthovanadate (SOV) presented no significant improvement at 3 mg/kg SOV but had significantly attenuated neuronal cell death in hippocampal CA1 region, improved neurofunction, and reduced brain edema after SAH at 10 mg/kg SOV. Thus, it could be debated that the toxicity of vanadium could be dependent on not just the dose or dosage, but on the duration of exposure, the route of exposure, the mechanism of action as well as the condition of the animals, whether in disease state or not. These findings are comparable with our observations because some aspects of the pathogenesis of brain damage or injury induced by hydrocephalus are very similar to those that occur in cerebral ischemia (stroke) and brain trauma (27).

## Conclusion

In this study, vanadium administration protected the hippocampal pyramidal cell layer as well as improved memory and spatial learning in juvenile hydrocephalic mice although the mechanism by which it acts in the hydrocephalic condition is unknown. This observation was in a dose-dependent manner, with the best recovery at 3 mg/kg; a widely dose reported to induce toxicity under normal, non-diseased conditions. Therefore, it could be argued that the vanadium-induced toxicity reported in literature were noticed in animals with normal physiologic conditions while the hydrocephalic state tempered the neurotoxic effects of the high dose. Neurodegenerative conditions could alter the mechanism through which vanadium may induce toxicity at the reported doses and extremely low doses of vanadium in these conditions might be too insignificant to produce any ameliorative effects or cause any significant effect in the signaling pathway. This study is the first investigation exploring the use of vanadium for hydrocephalus treatment, therefore, we recommend that mechanisms of vanadium action in hydrocephalus be further investigated. Biochemical studies may also be explored to find out the changes that occur as a result of vanadium administration in hydrocephalic states.

## Data availability statement

The original contributions presented in the study are included in the article/supplementary material, further inquiries can be directed to the corresponding author.

## Ethics statement

The animal study was reviewed and approved by the Ethical Review Board of the University of Ibadan UI-ACUREC/026-0421/20.

## Author contributions

OF-A: conceptualization, funding, data curation, project administration, investigation, writing—review and editing, and supervision. FO: conceptualization, funding, data curation, project administration, investigation, and writing—review and editing. JO: funding, data acquisition, writing—original draft, and investigation. JOO: funding, writing—review and editing, and validation. MS: writing—review and editing and validation. All authors contributed to the article and approved the submitted version.
